# Data extraction and comparison for complex systematic reviews: a step-by-step guideline and an implementation example using open-source software

**DOI:** 10.1186/s13643-023-02322-1

**Published:** 2023-12-01

**Authors:** Mohamed Afifi, Henrik Stryhn, Javier Sanchez

**Affiliations:** 1 Department of Animal Wealth Development, Biostatistics Section, Faculty of Veterinary Medicine, Zagazig University, Zagazig, Ash Sharqia Governorate 44519 Egypt; 2 Department of Health Management, Atlantic Veterinary College, University of Prince Edward Island, Charlottetown, PEI C1A 4P3 Canada

**Keywords:** Data extraction, Database, Guideline, Complex, Systematic review, Epi Info, R

## Abstract

**Background:**

Data extraction (DE) is a challenging step in systematic reviews (SRs). Complex SRs can involve multiple interventions and/or outcomes and encompass multiple research questions. Attempts have been made to clarify DE aspects focusing on the subsequent meta-analysis; there are, however, no guidelines for DE in complex SRs. Comparing datasets extracted independently by pairs of reviewers to detect discrepancies is also cumbersome, especially when the number of extracted variables and/or studies is colossal. This work aims to provide a set of practical steps to help SR teams design and build DE tools and compare extracted data for complex SRs.

**Methods:**

We provided a 10-step guideline, from determining data items and structure to data comparison, to help identify discrepancies and solve data disagreements between reviewers. The steps were organised into three phases: planning and building the database and data manipulation. Each step was described and illustrated with examples, and relevant references were provided for further guidance. A demonstration example was presented to illustrate the application of Epi Info and R in the database building and data manipulation phases. The proposed guideline was also summarised and compared with previous DE guidelines.

**Results:**

The steps of this guideline are described generally without focusing on a particular software application or meta-analysis technique. We emphasised determining the organisational data structure and highlighted its role in the subsequent steps of database building. In addition to the minimal programming skills needed, creating relational databases and data validation features of Epi info can be utilised to build DE tools for complex SRs. However, two R libraries are needed to facilitate data comparison and solve discrepancies.

**Conclusions:**

We hope adopting this guideline can help review teams construct DE tools that suit their complex review projects. Although Epi Info depends on proprietary software for data storage, it can still be a potential alternative to other commercial DE software for completing complex reviews.

**Supplementary Information:**

The online version contains supplementary material available at 10.1186/s13643-023-02322-1.

## Background

Data extraction (DE) is one of the most labour-intensive, time-consuming and error‐prone steps of systematic reviews (SRs) [1]. The validity of the SR findings depends on the accuracy and completeness of the data collected from the included studies [2, 3], and as a result, a rigorous and systematic approach to DE is needed to ensure an effective and appropriate DE. During DE, reviewers locate and extract data from the manuscripts and enter them into specifically designed DE tools. These data could encompass information about the methods, participants, settings, interventions, outcomes, results and investigators of the studies included in the review [4].

Seminal recommendations for DE have been made by different SR organisations, mainly the Joanna Briggs Institute [5] and Cochrane [6]. Besides reviewing different methodological aspects of DE, e.g. the development and pilot testing of the DE forms [7–9], errors from flaws in the DE process have been evaluated [10–13]. Additionally, some instructions for DE have been posted on different web pages (e.g. [14–16]), collectively contributing to a substantial improvement in the DE methodology.

The guideline of data extraction for complex meta-analysis (DECiMAL) [17] was mainly focused on considerations of DE concerning the subsequent meta-analyses, providing relatively little information on the practical and technical aspects of the DE. Moreover, in complex SRs, different meta-analytic techniques can be applied depending on the review’s objective. A previous eight-step data extraction and management guideline was centred on the Systematic Review Data Repository (SRDR) software [3], which might limit its application to other DE software. Despite the previous rigorous recommendations and guidelines, comprehensive practical information to plan and set up a database for DE and compare the extracted data between reviewers in complex reviews is still lacking.

Various DE software has been specifically developed to extract data from articles included in SRs, such as Covidence [18], EPPI-Reviewer [19], DistillerSR [20], Doctor Evidence [21], RevMan [22] and SRDR [3]. General DE software, including Access, MySQL, EpiData and Epi Info [23], has also been adopted to extract data in SRs. DE software generally spans a broad spectrum of complexity, from simple spreadsheets to more advanced databases [6]. Some are freely available, such as RevMan, SRDR and most of the general DE software, while subscription fees are needed for others. Moreover, some DE software is web-based, e.g. SRDR, whereas others can have optional internet access, e.g. Epi Info and RevMan, or work in a completely offline environment, e.g. Access. A detailed comparison of all DE software is beyond the scope of this paper; however, the available funding, complexity and size of the review, as well as the number and locations of the reviewers, need to be considered when deciding on the software of choice [3, 24].

The simplest and most common approach has traditionally been to extract the data directly into flat-file databases (e.g. Excel and Google Forms), which contain single or multiple self-contained tables of data [24, 25]. The flat-file databases can accommodate the data structure of simple SRs; however, their use can be challenging in complex reviews, which can address several linked research questions and/or multiple interventions and/or outcomes [26, 27]. Extracting and managing large and complex datasets with multiple dependencies (i.e. more than one effect size nested within a single study) would be more efficient using relational databases (e.g. Access), where data are typically structured across multiple connected tables. Moreover, identifying and resolving disagreements between pairs of reviewers may require excessive manual work when extracting data in flat-file databases [24]. Therefore, relational databases could be a better alternative for extracting and comparing data; however, more guidance on their implementation is needed.

In practice, DE encompasses a series of steps from planning and deciding on the data items that need to be captured until the data is ready for export to statistical software for analysis. Although there has not been any evidence suggesting that using a standard guideline for DE leads to less biased SR findings, it is still imperative to ensure that SRs follow an explicit methodology to be systematic, transparent and reproducible. A detailed guideline may also help advance the methodology in this area, and future SR teams can learn from previous ones. Moreover, novel software applications will give reviewers more flexibility in selecting the DE software that best suits the needs of their SR projects.

This article aims to develop a step-by-step practical guideline for DE with particular emphasis on planning and creating databases for complex SRs and to illustrate the application of Epi Info supplemented with two R libraries in DE using a worked example adapted from an ongoing SR project.

## Methods

**Fig. 1:**
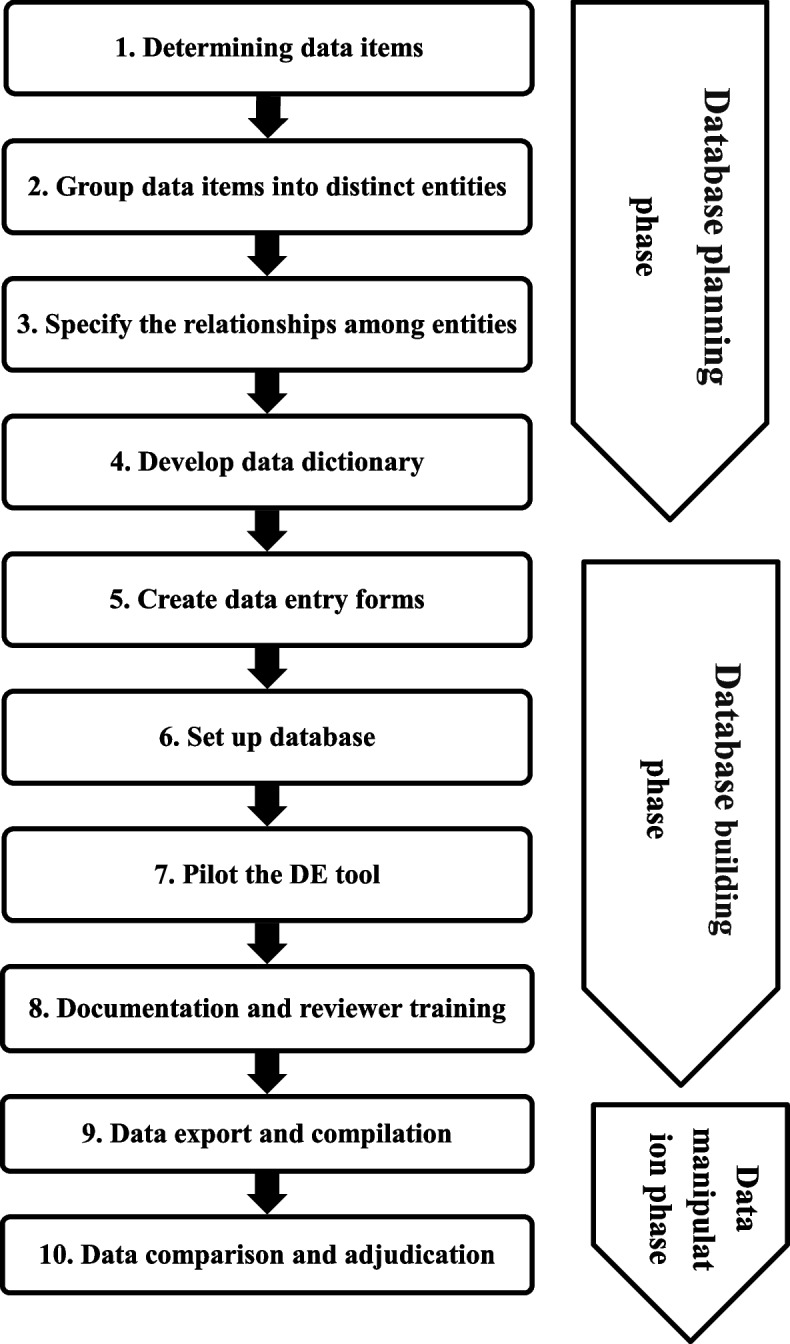
Flowchart of the DE steps, Epi Info was applied in Steps 5 and 6 of the database building phase, and R libraries were applied in the data manipulation phase (Steps 9 and 10)

This guideline comprises three main phases, database planning and building and data manipulation; each phase consists of consecutive steps, preferably done in the order listed (Fig. 1). Throughout, input is often needed from content experts, methodologists and statisticians on the outcome(s) data, other data pertinent to the research question, and data needed to evaluate the risk of bias [28]. Expertise in designing and setting up relational databases and data management can also help in the database planning and building phases. The team of content and methods experts, statisticians and database developers is referred to hereafter as the development team.

We also compared our proposed guideline with previous DE guidelines. A glossary of terms used in this manuscript is provided in Supplementary Table S1.

### Database planning phase

A database should be designed and planned from the outset to ensure consistency and efficiency in how data are extracted [29]. In this phase, the development team plans and drafts a preliminary design for the database. Designing the database could start from scratch, or databases designed for reviews on topics related to the review subject could be used.

#### Step 1: determine data items

Generally speaking, the data collected for SRs should describe the included studies, facilitate the risk of bias and GRADE assessments and enable meta-analyses. In this step, the development team addresses the following question: which data should be collected to answer the review question(s)? Previous knowledge of the topic area, a sample of key eligible articles and/or previously conducted SRs on the same or related topics can help identify pertinent data items. Data items can be dropped or modified, and additional data items can be identified when piloting the DE tool (Step 7); however, the review team needs to be updated with any changes.

Some DE software, e.g. RevMan [22], has the data items needed for bias assessment already built-in. The development team has to decide whether data items needed for bias assessment would be implemented in the DE tool, or standalone tools for bias assessment, such as the Excel ROB2 [30, 31] and the Access ROBINS-I [32, 33] would be used. Data items required for assessing the quality of the body of evidence using the GRADE approach, such as those needed to evaluate the comparability of the populations, interventions, comparators and outcomes of the studies forming the body of evidence to the target population (i.e. indirectness) [34] also have to be determined.

Different meta-analysis methods might be required to answer the review question(s); for instance, if the reviewers aim to identify sources of effect size variation across studies, more data items would be needed than if the aim is only limited to estimating an overall summary of effect sizes [35].

#### Step 2: group data items into distinct entities

The identified data items need to be logically grouped according to their relevance and position in the hierarchy into one or multiple entities, which would be translated into database tables in the following database-building phase. Entities, in database terminology, represent the principal data objects about which data needs to be collected [36].

**Fig. 2:**
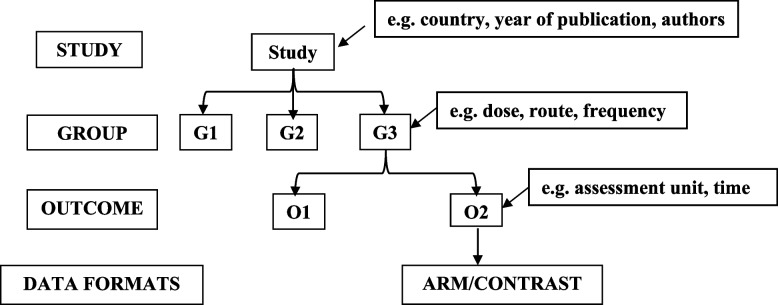
The tree diagram illustrates the hierarchical organisation of the data, including 5 entities arranged from top to bottom as STUDY, GROUP, OUTCOME, ARM and CONTRAST, along with their corresponding data items

In this step, the development team has to address this question: What would each row in the dataset represent (a study, a report (trial), or an outcome)? [29]. The organisational structure of the entities can be depicted using a simple tree diagram (e.g. Fig. 2), in which the root entity (top of the hierarchy) captures the data that only occur once in the article, e.g. study characteristics. Branch entities are then added to capture data repeated throughout the article due to multiple outcomes and/or interventions [25]. When more than one outcome value are extracted from each study, the resulting data will form a hierarchical (clustered) structure with studies at the top level. Such multiple outcome values may come from multiple interventions, other within-study subgroups or genuinely different outcomes [25, 28]. In Fig. 2, data items related to the intervention, including dose, route and administration frequency, are assigned to the GROUP entity; hereafter, entities’ names will be capitalised throughout the text. The database structure described herein is for DE purposes only; however, the resulting dataset can be wrangled into different formats for analysis [29].

#### Step 3: specify the relationships among entities

**Fig. 3:**
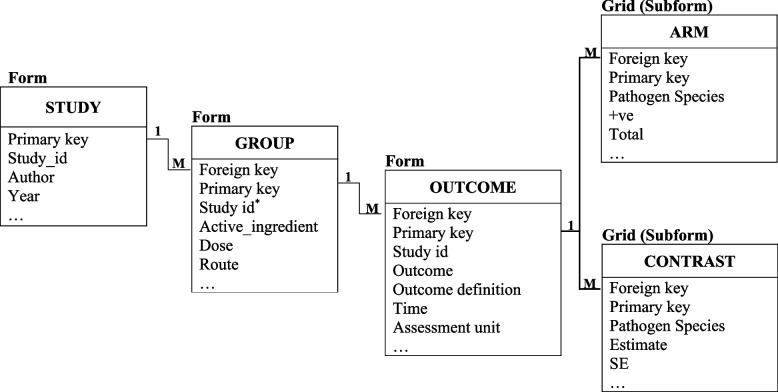
The full ER diagram shows the relationships among the different entities in the database. Each box symbolised a single entity corresponding to an Epi Info form, except the ARM and CONTRAST, which were constructed as grids (i.e. table-like data entry fields) and added to the main form. The data items were listed within each entity. The lines with ‘1’ and ‘M’ markings show the 1:M relationships among the entities. The primary and foreign keys are indicated as GlobalRecordId and FKEY in Epi Info, respectively

Each pair of entities can be connected through one-to-one (1:1), one-to-many (1:M) or many-to-many (M:M) relationships, depending on how each instance (a particular occurrence or entry) in the first entity relates to instance(s) in the second entity [36]. However, the relationship of primary importance for the hierarchical structure is the 1:M, where each instance in the higher-level entity can connect to many instances in the lower-level entity. Relationships among the different database entities can be depicted using an entity-relationship (ER) diagram where each entity is represented as a rectangle with the entity name written inside (e.g. Fig. 3).

Identification (key) variables, primary and foreign keys connect entities together, where the primary key uniquely identifies each row in the higher-level entity, and the foreign key matches the primary key record(s) in the lower-level entity [37]. The primary and foreign keys can be indicated in the ER diagram (e.g. Fig. 3). Existing or new automatically generated data items can be used as identification variables [38].

#### Step 4: develop a data dictionary

A data dictionary is a document that describes entities, their corresponding data items and the database structure (ER diagram) [29, 39]. Data dictionaries are more comprehensive than codebooks; they include explanatory notes about the database structure, and the information describes variables’ names, labels, types, formats, lengths and other special requirements (e.g. read-only, optional or mandatory) [40].

Following a consistent manner for naming the variables, e.g. camelCase or snake_case, makes them easily recognised when used in statistical software [29]. Using clear and simple language when wording variables’ labels, particularly when they form questions, would avoid confusion and facilitate the reviewers’ learning. More guidance on phrasing the data items and developing codebooks for SRs can be consulted [3, 41]. The categories of different variables should be predefined in an exhaustive and non-overlapping (i.e. mutually exclusive) way [41]; yet, for some variables, a complete list of categories cannot be anticipated. Creating separate lists of categories for such variables is recommended to add new or missed categories flexibly. An informative and well-structured data dictionary helps simplify the subsequent steps of the database building phase, ensures consistent responses among reviewers [41, 42] and enables the implementation of different data entry checks (Step 5). The variables should be listed in the same order as they would appear in the data entry forms.

### Database building phase

The database building phase forms the backbone of the DE guideline, where the conceptual database design gleaned from the planning phase turns into a physical database. Each entity turns into a database table, in which data are stored; each data item becomes a variable in this table, and data entry forms are also created in which data are keyed.

#### Step 5: create data entry forms

The data entry forms are the front interfaces that directly communicate with the reviewers; therefore, customisable and user-friendly forms are preferable. Data entry fields are created in each form, where reviewers enter data for individual data items. Generally, well-designed forms help minimise errors from miskeying or misclicking and reduce the time and effort spent extracting data [43]. Specifically, when the order of the forms and data entry fields closely follows the reporting flow of the information in the articles, they become easy to locate, and the number of cross-form moves is reduced. Relevant fields can also be logically grouped with suitable headings [6, 43]; for example, breed, age, inclusion and exclusion criteria can be gathered in one section of the form, so all information about the participants’ characteristics can be entered at once. A well-structured data dictionary (Step 4) minimises the time spent creating the forms [44] by directly guiding the development team to the appropriate field types (e.g. text, numeric, or dropdown lists) and other needed details.

Moreover, quality control checks, such as value range, field type and logic checks, help ensure compliance with data entry rules and reduce the likelihood of entry errors [44]. The value range checks are used for numeric fields with permissible ranges of answers, while field type checks verify that the data entered in a field are of the correct type; for instance, a decimal number will not be allowed in an integer-type field. Finally, logical relationships between fields can be set using if statements combined with conditional expressions (logic checks) to ensure logically consistent answers. For example, when the field “Nature of the infection” is answered as “Natural”, filling in the field “Challenge bacterial dose” gives an error message. Invalid answers for text fields can be much reduced using dropdown lists, even when permitting a free-text answer for an “other” category. Free-text fields can also be used to collect additional comments and capture direct verbatim quotes from the study whenever possible to support answers that can imply judgements in other fields [3].

#### Step 6: set up the database

In contrast to the flat-file databases, the DE tools based on relational databases comprise multiple structurally related tables where the data entered by reviewers reside [24, 39]. The relational database allows tables to be connected to each other using primary and foreign keys, as explained in Step 3. The user guide or manual of the software on which the development team decides must be consulted for setting up the database.

#### Step 7: pilot the DE tool

Testing the initial version of the DE tool on a small set of eligible studies would help identify any entry difficulties such as (1) the tool is not working properly (e.g. program glitches), (2) improper storage of the data, (3) omission of the logic or range checks, (4) incorrect labelling of variables or categories of dropdown lists and (5) missing relevant data items [3, 7, 43]. Although previous literature did not specify a particular number of studies needed to test the DE tool, a purposive sample of studies with one or multiple outcomes whose data are reported in different ways is recommended [16].

Quantifying the agreement between reviewers during the piloting process and postponing the extraction until reaching a satisfactory agreement level have also been reported, albeit no specific agreement thresholds were recommended [45, 46]. However, the piloting might need to be iteratively repeated until no major changes in the tool are needed. The review team, including reviewers, statisticians and content experts, are encouraged to participate in piloting the DE tool. Problems with the tool may still surface after pilot testing; therefore, the review team needs to be notified of any further changes.

#### Step 8: documentation and reviewer training

Detailed instructions on filling in the data fields and navigating among forms will increase the consistency of the extracted data between reviewers. We also advocate including illustrative examples in the tool manual to help reviewers learn and understand the data fields.

Training acquaints reviewers with the forms and helps solve any issues that may arise during the extraction [6, 7]. The training can be organised as a tutorial using a purposively selected sample of eligible studies. The involvement of the entire review team in training would allow for a comprehensive discussion between data extractors, clinicians and methodologists. Each data item should be carefully described during the training, and none should be overlooked or considered obvious.

### Data manipulation phase

In this phase, after the reviewers extract data from the studies included in the review, the data stored in the database tables are exported and combined into a single file. Then, the data need to go through some manipulation processes. In this phase, we assume that two reviewers would extract data independently from the same set of studies using two identical copies of the DE tool.

#### Step 9: data export and compilation

For each reviewer, the captured data are often individually exported from each database table and combined into a single data file. Even though data are exported as separate datasets, they can still be assembled using the identification (key) variables to make up a complete dataset. Exported datasets can be either combined through side-by-side merging using primary and foreign keys or concatenation, where one dataset is put at the end of another. Different statistical software can accomplish the data compilation procedures [47].

#### Step 10: data comparison and adjudication

The end product of independent double data extraction is two datasets, one for each reviewer, that need to be compared to identify any discrepancies. Discrepancies are due to an unmatched number of observations (rows) or different values of observations per se. We do not advocate postponing the data comparison until after data have been extracted from all studies. Instead, we recommend more frequent comparisons using subsets of studies to limit the need to go back to the articles and re-extract data due to systemic errors in interpreting the data items. Further, a comparison of the entire dataset (i.e. all variables together) might not be manageable due to the different hierarchical levels where variables are recorded; therefore, splitting it into subsets of variables might be more feasible.

Data adjudication is when decisions are made to solve disagreements, and subsequent data edits occur. A third reviewer is often called upon to resolve disagreements, and the reconciliation procedure between reviewers should be reported [7].

## Implementation example and software application

In this section, we describe the adoption of the proposed DE guideline to an ongoing complex SR project, which encompasses five research questions addressing the efficacy of different antimicrobial treatments on three outcomes: incidence, prevalence and cure of intramammary infections [48]. Epi Info software was used for the database building phase, and two R libraries were needed for the data manipulation phase. Data were independently extracted from eligible studies by two reviewers following the standard double DE approach. The steps of the data manipulation phase were illustrated using the data extracted from Bradley et al. [49] study, one of the eligible studies for the project. In this article, the outcome data were reported in both arm- and contrast-based formats; additionally, different subtypes of intramammary infections caused by different pathogen species were reported. Such reporting of several effect sizes per study is often referred to as effect size multiplicity, which occurs when the primary studies report multiple effect sizes coming from multiple analyses of the same outcome, or when an outcome is measured/assessed at multiple time points or in different units/scales based on data from the same participants [50, 51].

### Database planning phase

 In this phase, a preliminary design for the database was constructed. No specific software was required; only standard text processing software was needed to create the data dictionary and sketch the ER diagram.

#### Step 1: determine data items

This step was accomplished during the protocol development, consulting previous relevant SRs and other SR protocols. A group discussion involving a multidisciplinary development team with clinical (dairy cows) and methodological expertise took place to decide on the list of the relevant data items. The development team decided against incorporating the bias assessment items in the DE tool; however, some data items describing the interventions and the participants in the included studies were needed for the GRADE assessment.

#### Step 2: group data items into distinct entities

The selected data items were assigned to six entities: STUDY, TRIAL, GROUP, OUTCOME, ARM and CONTRAST. To ease the presentation, the entity TRIAL and the trial-level data items were dropped, and only 5 entities were depicted in Fig. 2.

#### Step 3: specify the relationships among entities

The ER diagram (Fig. 3) presents the structural organisation of the different entities and their relationships. A study must contain two or more intervention groups to be eligible for inclusion; in the same way, each intervention group could be linked to at least one of the three outcomes of interest (cure, incidence, prevalence). Therefore, a 1:M relationship was configured between the STUDY and GROUP and the GROUP and OUTCOME entities. Then, each outcome could be reported in arm- and/or contrast-based data formats so that two more entities were constructed, ARM and CONTRAST, where each was then connected in 1:M relationships to the OUTCOME entity.

Initially, the development team decided to use natural (already existing data items) as primary keys for the different entities, e.g. study ID and active ingredient. However, Epi Info automatically generates surrogate keys (i.e. meaningless in the context of the SR), which seemed more practical to use [52].

#### Step 4: develop a data dictionary

For each identified data item, name, type and allowed values were specified in the data dictionary; all entities and their corresponding data items are available in the Additional file 1, Section 1. Exhaustive lists of all possible antimicrobials and subtypes of infection (due to different pathogens) could not be prespecified beforehand. Alternatively, allowing reviewers to use free-text data would lead to variations in the entered data (e.g. Staphylococcus aureus, Staph. aureus and S. aureus), complicating the subsequent data comparison between reviewers. Therefore, preliminary lists of antimicrobials and infection subtypes were initially used and continuously updated with newly encountered values of antimicrobials and infection subtypes. In the database built for this SR project, we did not name the variables in a consistent way; however, the development team was responsible for the analysis, so they were fully aware of all the variables’ names and indications.

### Database building phase

Epi Info was the most suitable software based on the available time for the review project and the technical skills of the development team. Epi Info version 7.2.4 was used for developing the DE tool. The tool has a project file (.prj), which holds the data entry forms, and an Access file (.mdb), which contains the database tables and the entered data. Both files need to be located in the same directory on the computer for the Epi Info to execute. Details about the Epi info DE operational requirements are described in the Additional file 1, Section 2.

#### Steps 5 and 6: create data entry forms and set up the database

Since creating the forms and defining the database tables occur simultaneously in Epi Info, we illustrate these two steps together. The default Epi Info project has one form with one page where data entry fields are added. Because each form has only one table in the database, three forms, study, group and outcome (Appendix), were created and their corresponding tables were linked to each other in a 1:M fashion using the “Relate” button. In this way, each entry in the study form can have many corresponding entries in the group form, which in turn can have many entries in the outcome form. The study was the top-most (root) form, which opens first when reviewers run the Epi Info project file.

The relationship between the forms reflects the relationship between their corresponding tables at the backend such that a single instance of the primary key (“Globalrecordid” in Epi Info) in the study table connects to multiple instances of the foreign key (“FKEY” in Epi Info) in the group table when data of multiple intervention groups for the same study are extracted (Fig. 3). The ER diagram (Fig. 3) illustrates that the outcome table is linked through 1:M relationships to the arm and contrast tables to capture the arm- and contrast-based data reported for each outcome, respectively. Instead of creating other forms for the arm and contrast tables, grids which are dynamic table-like data entry fields, were added to the outcome form. The grid implements a sub-form with a corresponding table that links in a 1:M fashion to the table of the form in which the grid is created. Therefore, grids are practical in case of listing questions such as “List the infecting pathogen species, the number of positive, and the total”.

Data entry fields were then created in the forms; for example, the outcome form includes data fields to capture the outcome settings, e.g. the outcome per se (i.e. incidence, prevalence and cure), outcome definition, outcome assessment time (i.e. days post-calving) and unit (i.e. cow, and quarter). The data dictionary helped the development team select the appropriate field type and phrase the texts or questions describing the data needed to be collected. Navigation among forms was facilitated by using read-only mirror fields, which carry identifying variables from one form to the next. Check codes were also used to implement quality control checks and to help validate the entered data; see the user guide for available check codes [52].

#### Step 7: pilot the DE tool

Before starting the extraction of data from the eligible articles, three reviewers tested the initial version of the DE tool in a sample of five articles, which were purposely selected to include studies that reported results in arm- and contrast-based formats for more than one research question and two or more outcomes. We learned that involving field experts in this step would have been ideal. The DE tool created using Epi info was flexible for changes.

#### Step 8: documentation and reviewer training

A comprehensive manual, provided in the Appendix, was developed with detailed instructions on installing and executing the Epi info DE tool, filling in the different data fields and moving among forms. We also supplemented the manual with practical examples whenever needed to help guide the reviewers to extract the correct data. We arranged in-person and online sessions to introduce the review team to the tool and train them on extracting data using two eligible articles, which were deliberately selected to familiarise the extractors with the different ways the outcomes data were reported. The review team was kept abreast of any changes in the tool.

### Data manipulation phase

#### Step 9: data export and compilation

The data from the Epi Info DE tool were exported and saved as CSV files using a series of READ and WRITE commands, which were run in the command-line interface of Epi Info. Data were then read into R, and the inner_join function of the tidyverse R library was used to join each row of the parent (higher-level) table with the corresponding row(s) in the child table using the “Globalrecordid” and “FKEY” variables, resulting in a single data table. Codes for exporting 6 datasets from Epi info and subsequent data compilation in R are available in the Appendix.

#### Step 10: data comparison and adjudication

**Fig. 4:**
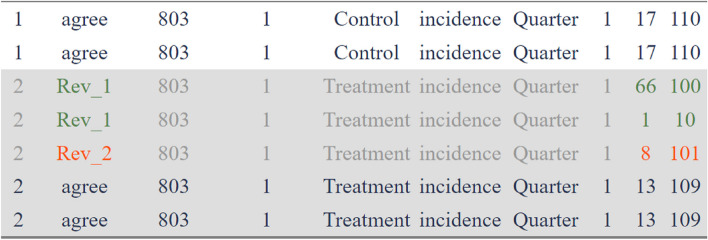
The output of the compareDF library. The colour schemes facilitate the recognition of the discrepancies and agreements between the two reviewers. A single cell is coloured if it has changed across the two datasets. The discrepancies in the values in the first and second reviewer datasets were coloured green and red, respectively. Cells that did not change across the two datasets are coloured blue

Different R libraries can facilitate the comparison of datasets extracted by pairs of reviewers, such as dataCompareR [53] and compareDF [54]. The compareDF library highlights the discrepancies using a colour scheme so they can be quickly sorted out (Fig. 4), and its output can be rendered in different formats such as HTML, XLSX and PDF. Moreover, differences due to rounding of numerical variables can be ignored by setting the tolerance argument to a user-specified absolute difference or ratio. Character tolerance is also available in other R packages for categorical data, which allows for ignoring case differences or other differences in the white space or a certain set of characters.

After data compilation, to compare the datasets extracted by two reviewers, each dataset was split into subsets of variables depending on the hierarchical level at which they were captured. Then, unique (distinct) rows in each subset were compared using the compare_df function of the compareDF library using matching variables. The difference in the number of observations of each dataset was also revised. Two datasets, “Rev_1” and “Rev_2”, and the R codes for data comparison were provided in the Appendix.

Disagreements were checked by the two reviewers, and a final decision was reached by discussion. The most difficult disagreements were reconciled in a group with a third reviewer. The data adjudication usually starts from the bottom (lower level) datasets concerning the hierarchical data structure, i.e. arm and contrast. The more complete dataset or the one extracted by the more experienced reviewer can be used as a template, and the other can be used to complement it. More details for fixing disagreements are provided in the Additional file 1, Section 3.

## Results

### Comparison with the previous DE guidelines

The steps and key messages of the proposed and Li et al. [3] guidelines are summarised in Table 1. Our guideline was developed to fit the SR objectives without focusing on a particular software application or meta-analysis technique. The proposed and Li et al. [3] guidelines agreed on the first two steps of determining and arranging data items; however, we emphasised identifying the organisational data structure and configuring the relationships among the data tables. Details about data manipulation and comparison procedures were additionally provided in the last two steps of our guideline.

**Table 1:** Summary of the steps and key messages of the proposed and the previous guideline of Li et al. [3]

Proposed guideline	Previous guideline of Li et al. [3]
**Step 1: Determine data items** • Identify the objective of the systematic review. • Identify the data items that are relevant to the research questions. • Use previous relevant reviews and eligible articles as a guide. • Determine how bias assessment data will be captured.	**Step 1: Develop outlines of tables and figures** • Develop outlines of the tables and figures that will appear in the SR beforehand.
**Step 2: Group data items into distinct entities** • Identify the hierarchal data structure. • Group the data items according to their level in the hierarchy. • Ensure that the entities are organised hierarchically, with the top-most entity capturing the data that only occur once in the article.	**Step 2: Assemble and group data elements** • Important characteristics that would modify the treatment effect or the association of interest should be collected. • Group data elements in the order in which they are usually found in study reports (e.g. starting with reference information, followed by eligibility criteria, intervention description, statistical methods, baseline characteristics and results).
**Step 3: Specify the relationship among entities** • Specify appropriate relationships among the entities. • When a data item at a higher-level entity is expected to correspond to many data items down the hierarchy, a 1:m relationship would best fit. • Determine the data items that will be used as primary and foreign keys. • Construct an ER diagram.	NE
**Step 4: Develop a data dictionary** • In addition to key messages reported in Steps 3 and 4 of Li et al. (2015) guideline [3], define variables’ names, labels, types, formats, lengths and other special requirements if needed. • Name the variables in a consistent way, so they can be easily recognised and used in statistical software. • The variables should be listed in the same order as they would appear in the data entry forms.	**Step 3: Identify the optimal way of framing the data abstraction item** • Ask closed-ended questions as much as possible. • Avoid asking a question in a way that the response may be left blank. Include ‘not applicable’, ‘not reported’ and ‘cannot tell’ options as needed. • Open-ended questions are useful when it is not possible to anticipate the different responses that may be given or when it is necessary to avoid leading the data abstractors by indicating permissible replies. • Remember that the form will focus on what is reported in the article rather than what has been done in the study. • Ask 1 question at a time to avoid confusion. • When a judgement is required, record the raw data (i.e. quote directly from the source document) used to make the judgement. • Record the data as provided in the source document to minimise the mathematical manipulations required during DE.
**Step 5: Create data entry forms** • The order of the forms and data entry fields needs to closely follow the reporting flow of the information in the articles. • Use quality control checks, such as value range, field type, and logic checks, whenever applicable.	**Step 4: Develop data abstraction forms** **• **Develop data abstraction forms using word processing software to serve as a guide for creating an electronic data abstraction form and a codebook. • Definitions and instructions helpful for answering a question should appear next to the question to improve quality and consistency across data abstractors. • The quality control checks were reported further later in Step 7.
**Step 6: Setup database** • Review the software’s manual or user guide to build and connect the database tables.	NE
**Step 7: Pilot the DE tool** • Take a purposive sample of studies with results reported in different ways. • Check for difficulties such as (1) forms are not working properly; (2) improper storage of the data; (3) omission of the logic or range checks; (4) incorrect labelling of variables or dropdown menu categories; and (5) missing relevant data items. • Reviewers, statisticians and content experts should be engaged in the piloting of the tool.	**Step 5: Set up and pilot-test data abstraction forms in the SRDR** • Develop a user manual with instructions, coding conventions, and definitions specific to the project. •Testing the DE tool should involve several persons abstracting data from at least 3 articles.
**Step 8: Documentation and reviewer training** • Develop a comprehensive manual with detailed instructions on filling in the data fields and navigating among forms. • Supplement the manual with practical examples to help reviewers understand the data items and extract reliable data. • The articles used in training should be selected to show a variety of data reporting. • The entire review team, including data extractors, clinicians, and methodologists, needs to be involved in this step. • Each data item should be thoroughly described.	**Step 6: Train data abstractors** • Training should include modules to familiarise the review team with the data system and data abstraction form. • Complete the general SRDR training modules. • Data abstractors should have a basic understanding of the clinical issues surrounding the topic, study design, analysis, and statistics. • Pay attention to details while following the instructions on the forms and the user manual. • Training sessions should take place at the project onset and intermittently over the course of the project.
The key messages reported in Step 7 of Li et al. [3] are included in different steps of the proposed guideline; for instance, double data extraction and comparison are covered in Step 10. The logic checks were also reported in Step 5.	**Step 7: Implement a quality assurance and control plan and monitor the progress** • We recommend having 2 data abstractors who work independently to collect data on the SRDR. • The Data Comparison Tool in the SRDR. • Create Logic checks • Monitor the timeliness of data abstraction and progress.
**Step 9: Data export and compilation** • Depending on the data structure, data processing, e.g. merging and concatenation, are needed to assemble separate datasets exported from the database into a single dataset.	**Step 8: Export and clean the data for analysis** • A specific subset of data can only be exported from SRDR. • Each worksheet contains data collected from 1 tab in the SRDR. • Data can be imported into statistical software for processing and analysis.
**Step 10: Data comparison and adjudication** • Split the dataset into subsets of variables, depending on the hierarchical level at which they were recorded. • More frequent comparisons and adjudications are better than waiting until data have been extracted from all studies.	• A tool for data comparison implemented in the SRDR software was described in step 7.

*NE*: a corresponding step does not exist

### Application of Epi Info in DE for systematic reviews

Features of Epi Info that support its application in the DE for simple and complex reviews, in particular, are listed in Supplementary Table S2. Broadly speaking, the DE tool developed using Epi Info is user-friendly, and the forms are flexible for arranging data entry fields and editing during piloting. Based on our learning experience, Epi Info does not require extensive programming skills for setting up the database relative to other database development software, e.g. MySQL. Linking the database tables allowed the tool to accommodate the data structure needed for our complex review. We also managed to directly import the bibliographic data from the reference management software (EndNote) into the Epi Info DE tool (i.e. prefilling), so there was no need to enter these data. Supplementation of the Epi Info DE tool with 2 R libraries tidyverse and compareDF was needed to facilitate the compilation, comparison and adjudication of the extracted data.

### Epi Info DE tool operational requirements

Reviewers without prior experience using DE tools developed by Epi Info found no difficulties entering data and browsing between the forms. The Epi Info DE tool works in an offline environment with minimum Windows, RAM and processor requirements; however, Access must be installed on local computers. Some technical issues were encountered while building and piloting the tool; however, they were all solved after consulting with the Epi Info help desk and the users’ community portal without subscription costs.

## Discussion

### Summary of main findings

This manuscript aimed to develop a guideline for DE in complex SRs, which can include more than one research question, multiple interventions and/or outcomes, regardless of the subsequent meta-analysis approach. The guideline includes 10 steps to help reviewers plan and build DE tools and compare the extracted data between reviewers. We emphasised determining the organisational data structure and setting up a database to accommodate such structure. We also focused on creating the data entry forms and tables and specifying inter-table relationships. The guideline applies equally to simple and complex reviews, albeit fewer entities and simpler data structures would be expected in the former.

Reviewers, especially those conducting complex reviews or dealing with effect size multiplicity, are tempted to extract all effect sizes reported in the included articles in a neat and organised manner. This approach can lead to an unmanageable amount of data to extract. For instance, 18 or 36 rows of contrast- or arm-based data, respectively, need to be extracted from a single study addressing the effect of two interventions (Treatment versus Control) on three outcomes (e.g. X, Y and Z), which are assessed at two different time points (e.g. 6 and 12 days) with overall and stratified results (e.g. A and B subgroups). The data dimensions substantially increase when the SR includes more than one research question and more than two interventions. Such complex situations are generally not unusual, particularly in animal health research reviews, where variation in reporting the results between studies is customary.

In complex reviews, using flat-file databases may lead to unnecessary repetition of entering data that occurs once in the study (e.g. publication year or the study design), increasing the likelihood of entry errors as well as the time spent extracting data, particularly when the number of studies is large. Alternatively, using separate (i.e. unrelated) tables for extracting such data risks the data integrity, as changes in one table might not be reflected in the other.

DE tools built using relational databases can store and organise data in related tables, avoiding data redundancy (i.e. entering repeated data) [17, 55, 56] and preventing the risk of inconsistent and outdated data. Therefore, relational databases can be the best choice for collecting data from primary studies with effect size multiplicity and/or in complex SRs.

### Comparison with previous guidelines

Our guideline broadly aligns with the previous 5-step guideline for setting up databases for clinical trials, which includes data collection, database conception (structure and organisation), database building, data validation and software application [57]. However, in our guideline, we provided detailed steps underlying each phase and pointed out the specifications of the DE for complex SRs.

The DECiMAL guideline is primarily focused on some aspects of the DE that relate to the subsequent meta-analysis [17]; however, we believe that a guideline needs to fit the objectives of the SR regardless of the meta-analysis methods that will be employed. Additionally, different meta-analysis methods can be applied in complex SRs encompassing more than 1 research question.

Compared to the guideline of Li et al. [3], we emphasised determining the hierarchical structure of the data, grouping data items into entities and configuring the relationships between the entities, which have no corresponding steps in the previous guideline. We focused on using the data dictionary instead of the codebook to comprehensively represent the data entities, their corresponding data items and the links between them using the ER diagram.

In the database building phase, the steps of creating the data entry forms and setting up the database were missing from the previous guideline [3]. However, some of the points covered in these two steps in our guideline were referred to in other steps of the previous guideline. Steps 5 and 6 of the previous guideline are similar but not identical to steps 7 and 8 of the proposed guideline.

In the data manipulation phase, we accentuated the different techniques that can be used for data compilation and comparison steps. The previous guideline relied on a built-in feature for data comparison available in some SR software, e.g. SRDR and Covidence, which allows for instant comparisons of data entered by reviewers upon the second extraction at each entered value. We, however, believe that this feature may introduce bias, mainly when the two reviewers are not blinded to each other, which is an inevitable practice. Nevertheless, evidence of such bias has not been reported in the literature. Discrepancy checking was previously applied using a database implemented in Access and complemented with Visual Basic modules and SQL scripts [58].

### The implementation example and our experience with using Epi Info for DE

The implementation example can be replicated using the online materials in the Appendix. The Epi Info DE tool was also provided for reviewers to use and adapt to other SR projects. Epi Info software has been widely used to collect and analyse public health data [59–61]; however, to our knowledge, its application for DE in complex reviews is novel. The absence of applications could be due to a lack of previous documentation describing the implementation of Epi Info features in DE for SRs. The lack of functionalities necessary to compare the extracted data between reviewers and fix discrepancies could also be a reason.

We learned that Epi Info could be easily configured to support the specifics of DE for simple and complex reviews, and complementing its functionalities with R libraries for data compilation and comparison makes it a potential alternative to other commercial software. The menu toolbar of Epi Info allows reviewers without programming experience to set up and customise the data entry forms [62]. Epi Info uses relational databases for creating and joining tables to accommodate the hierarchical data structure needed for complex reviews, avoiding data redundancy and ensuring data integrity. Additionally, its rigorous control over the data entry through specifying the data types and setting check codes reduces error possibilities and discrepancies between reviewers [61].

Our application of Epi Info adds to the software options available for DE in SRs. It could also elicit further comparisons between the general and SR-specific software.

## Limitations

This guideline was inspired by our ongoing systematic review, which focuses on assessing the efficacy of interventions. We conjecture that the proposed guideline can extend to other types of SRs; however, the feasibility of its adoption and further applications remains to be shown. Automation techniques for DE, extracting data from graphs or contacting corresponding authors to obtain individual participants data were not in the scope of this manuscript. We believe it is reasonable to characterise the application of Epi Info and R in DE for SR as promising, although we did not compare their performance with currently existing tools.

## Conclusion

We hope this guideline can help reviewers design and build DE tools for complex SRs. Applying this guideline as part of the routine SR can simplify the DE process, boost its consistency, reproducibility and efficiency and enhance the quality of the subsequent meta-analysis. Two open-access software, Epi Info and R, were used for creating the database, data compilation, comparison and adjudication. Epi Info can be used to create a tool for extracting data for complex SR projects; however, additional R libraries are needed to compile and compare the extracted data between reviewers and make the data edits after solving discrepancies. Complementing the Epi Info functionalities with R renders it a potential alternative to other commercial software.

### Supplementary Information

**Additional file 1.** Sections 1 to 3.

## Data Availability

The datasets used in the manuscript and the R codes are available on a publicly accessible website (see Appendix).

## Appendix

A blank Epi Info DE tool, R codes and other material of this manuscript were organised depending on the step they were mentioned and made available on GitHub (https://github.com/Afifistat/Data-extraction-guideline.git).
