# Supplementary material for: Data extraction and comparison for complex systematic reviews: a step-by-step guideline and an implementation example using open-source software
Source: Syst Rev. 2023 Dec 1;12:226. doi: 10.1186/s13643-023-02322-1 (PMC10691069; doi:10.1186/s13643-023-02322-1)
Supplement: Supplementary file 1 — Additional file 1. Sections 1 to 3. [file 13643_2023_2322_MOESM1_ESM.docx]

**Supplementary Materials for:**

**Data extraction and comparison for complex systematic reviews: A step-by-step guideline and an implementation example using open-source software**

**Authors:**

Mohamed Afifi^1,2*^, Henrik Stryhn^2^, and Javier Sanchez^2^

^1^Department of Animal Wealth Development, Biostatistics Section, Faculty of Veterinary Medicine, Zagazig University, Zagazig, Ash Sharqia Governorate, 44519, Egypt;

^2^Department of Health Management, Atlantic Veterinary College, University of Prince Edward Island, Charlottetown, PEI, C1A 4P3, Canada;

*Corresponding Author: Mohamed Afifi

E-mail: [MAAfifi@vet.zu.edu.eg](mailto:MAAfifi@vet.zu.edu.eg); [mafifi@upei.ca](mailto:mafifi@upei.ca)

**Table S1.** Glossary of terms

| **Term** | **Definition/ Explanation** |
| --- | --- |
| Flat-file database | A database that is designed around a single table, and it is often used in spreadsheet programs. |
| Relational database | A database that is composed of a collection of related tables. |
| Entity | An entity is a principal object about which data need to be collected; it can denote a person, a place, or a thing of informational interest. Entities would be the tables of the database. |
| Entity relationship (ER) diagram | A graph shows how the entities relate to each other. |
| Data dictionary | A document that describes the entities, their corresponding data items and the database structure (ER diagram). |
| Data integrity | Because of the relationships between tables in relational databases, modification or deletion of a row in one table is reflected in other linked tables. Maintenance and assurance of such dependencies between tables is referred to as data integrity, which is vital to ensure that data in a database are correct and consistent. |
| Effect size multiplicity | Occurs when the primary studies report multiple effect sizes coming from multiple analyses of the same outcome, measuring/assessing the same outcome at multiple time points or in different units or scales based on data from the same participants |
| Arm-based data | Refers to raw data for each treatment arm/group of the study; for instance, for a binary outcome, it can be counts, absolute odds or risk. |
| Contrast-based data | Represents the relative treatment effects between arms, e.g. raw or adjusted odds rations |

**Table S2.** Epi Info features that support its application in DE for complex SR.

| **Features** | **Application** |
| --- | --- |
| Public domain software | - Epi Info DE tools suit SR projects with no or limited funding. |
| Optional internet access | - DE using Epi Info tools can be completed without an internet connection, and reviewers can work independently. - The Web and Cloud version of Epi Info ([1](#_ENREF_1)) would allow multiple reviewers to enter data simultaneously, providing online (cloud) data storage. |
| Customisable and user-friendly data-entry forms | - Epi Info DE tool requires minimal reviewer training to use effectively. |
| Minimal programming skills | - Reviewers without previous programming experience can set up their DE tools. |
| Check codes | - It is a feature of Epi Info that allows users to customise the data entry process. It is useful to check for errors during the data entry process, to do automatic calculations in fields, and to skip over parts of the forms if certain conditions are met. Therefore, it can help in the data validation and reduce discrepancies between reviewers. |
| Relational databases | - Avoid data redundancy. - Maintain and track the relationships between different entries. - Keep data integrity. |
| Technical support | - The Epi Info support and development team helps troubleshoot technical problems and provides swift solutions. |

# DCT Data dictionary

This section presents entities and their corresponding variables in Tables S3-S8.

## Table S3. Summary of the data items of the study entity

| **Name** | **Type (format)** | **Caption (Special info)** |
| --- | --- | --- |
| Study id^*^ | Integer (###) | Read-only. |
| Study General Info | Heading | Non-fillable field. |
| Year of Publication^*^ | Integers (####) | Read-only. |
| Type of Publication | List | Conference proceedings/Journal article |
| Country of cows**^✝^** | List | Must-enter  The place where the study was conducted (not the origin of the authors). |
| Author^*^ | Text | Read-only. |

^*^Imported directly from the bibliographic management softwares (EndNote).

**^✝^** The list of values of this field is continuously updated with encountering new values.

## Table S4. Summary of the data items of the trial entity

| **Name** | **Type (format)** | **Caption (Special info)** |
| --- | --- | --- |
| ID | Heading | Non-fillable label |
| Study id expo**^M^** | Integer | Mirror variable |
| Trial order | Integer | Order of the trial based on the sequence they were reported in the study. |
| Study design | List | (RCT/NRSI) |
| Participants_info | Heading | Non-fillable label |
| Total no of farms selected for inclusion | Integer |  |
| Total no of cows selected for inclusion | Integer |  |
| Total no of quarters selected for inclusion | Integer |  |
| Breed | List | (Holsteins/ Jerseys / Holst-Jerseys /Mix/ Others/ Not-reported) |
| Type of exposure | List | (Natural IMI/ Experimental Challenge /Natural- Experimental Mix) |
| Challenge Bacterial Sp. | List | Skip if Natural IMI  (Link to pathogens table) |
| Challenge dose | Float (##.##) | Skip if Natural IMI |
| Bacterial Conc.(CFU/ml) | Float (##.##) | Skip if Natural IMI |
| Methodological quality items | Heading | Non-fillable label |
| If the data have a hierarchical structure, have the authors accounted for clustering in their analysis? | List | (Yes, No, Data do not have a hierarchical structure) |
| Was the analysis done as intention to treat? | List | (Yes, No, No information) |
| Do you consider the methodology used for measuring the outcome inappropriate? | List | (Yes, No) |
| Was an a priori sample size calculation reported? | List | (Yes, No) |

**^M:^** The mirror variable reflects the trial order in the subsequent children forms.

## Table S5. Summary of the data items of the group entity

| **Name** | **Type (format)** | **Caption (Special info)** |
| --- | --- | --- |
| ID | Heading | Non-fillable label |
| Study id expo**^M^** | Integer | Mirror variable |
| Trial expo**^M^** | Integer | Mirror variable |
| AB info |  | Non-fillable label, |
| Group order | Integer |  |
| Active ingredient**^✝^** | Integer | -Ve cont/ Placebo/Enro/Tylosin/ … |
| Active_ingredient_expo | String | Read-only variable, mirror exposing the selected active ingredient which is used in combination with group_order to create group_expo. |
| Concentration | Float (##.##) | Skip if either -Ve cont or Placebo. |
| Concentration unit | List | IU/ mg/ml |
| Dose | Float  (##.##) | Quantity injected |
| Route | List | Intra-mammary/IM/ SC/IV/Topical/Oral |
| Frequency of administration | Integer |  |
| Interval between injections | Integer | e.g. 1, 2, 3, …, 14 days |
| Administration Regime | List | Selective/Blanket |
| If selective, what were the selection criteria? | Integer | Skip if Blanket  Petrifilm/SCC/Previous mastitis history |
| AB preparation | Integer | Commercial/Experimental |
| Trade name | String | Skip if Experimental |
| Manufacturer Company | String | Skip if Experimental |
| TS info |  | non-fillable label |
| TS_active ingredient | List | bismuth subnitrate |
| Ts_Conc (%) | Integer | e.g. 65 |
| Ts trade_ Name | String | e.g. Teatseal |
| TS_ Company | String | e.g. Zoetis |
| Role in PICO | Heading | non-fillable label |
| PICO1-2 | List | Control/ Treatment/ Not-used |
| PICO3-4 | List | Control/ Treatment/ Not-used |
| PICO5 | List | Control/ Treatment/ Not-used |

**^✝^**: The list of values of this field is continuously updated with encountering new values.

## Table S6. Summary of the data items of the outcome entity

| **Name** | **Type (format)** | **Caption (Special info)** |
| --- | --- | --- |
| ID | Heading | Non-fillable label |
| Study id expo^M^ | Integer | Mirror variable |
| Trial expo^M^ | Integer | Mirror |
| Group expo^M^ | String | Mirror, automatically created variable exposing both the order of the corresponding active ingredient of the group, e.g. 1_Clox i.e. the 1st cloxacillin group |
| Outcome_info | Heading | non-fillable label |
| Outcome | List | Cure/Incidence/Prevalence |
| Outcome definition | String | Multiline entry |
| Detection Method | Integer | Culture/SCC/PCR |
| COV (cut-off value) if SCC (1000 cells/ml) | Integer | Skip if Culture or PCR  e.g. 200 or 150 |
| unit | Integer | Cow/ Quarter |
| Time_info | Heading | non-fillable label |
| Time | Integer | Time |
| days_post_calving | Integer | e.g. 7, 14 or 20 |

## Table S7. Summary of the data items of arm entity

| **Name** | **Type (format)** | **Caption (Special info)** |
| --- | --- | --- |
| pathogen**^✝^** | List | Pathogen |
| +Ve | Integer |  |
| totals | Integer |  |

**^✝^**: The list of values of this field is continuously updated with encountering new values.

## Table S8. Summary of the data items of the contrast entity.

| **Name** | **Type (format)** | **Caption (Special info)** |
| --- | --- | --- |
| Comparison group | List | -Ve/ cont/Placebo/Enro/Tylosin/ … |
| Estimate type | List | OR/ RR/IRR/HR |
| Estimate scale | List | Linear/ln |
| Estimate | Float |  |
| SE | Float |  |
| CI level | List | 95%/99% |
| LCI | Float | LCI |
| UCI | Float | UCI |

## Table S9. List of countries where studies were conducted

| **Country*** |
| --- |
| Argentina |
| Australia |
| Brazil |
| Canada |
| China |
| Denmark |
| France |
| Germany |
| Hungary |
| India |
| Iran |
| Ireland |
| Israel |
| Italy |
| Mexico |
| Netherlands |
| New Zealand |
| Norway |
| South Africa |
| Turkey |
| UK |
| US |
| ……. |

*The list of countries will keep growing and extending once more articles are entered.

# Epi Info DE tool

## Epi Info DE tool operational requirements

Epi Info is freely accessible through the Centers for Disease Control and Prevention, where it has been developed and maintained. However, its application depends on one of two proprietary software: Microsoft Access or SQL server database. Broadly speaking, using open-access tools to accomplish different SR steps (i.e. open evidence synthesis) would allow for better reproducibility and minimise subjectivity as well as bias ([2](#_ENREF_2)). Moreover, using Epi Info to develop DE tools can help SR teams with limited resources avoid the costs of software subscriptions, hiring database developers, and using servers.

The source codes of some of the editions of Epi Info are made publicly available (i.e. open-source) ([3](#_ENREF_3)). This feature can attract more developers, allowing for more enhancements and adaptations of its functionalities ([3](#_ENREF_3), [4](#_ENREF_4)).

Although we used the Windows operating system, a cross-platform design for Epi Info has been developed to allow its execution in Linux and Mac OS ([5](#_ENREF_5)). Epi info DE tools can also operate with or without an internet connection; this feature is particularly beneficial in locations or countries where internet access might be a barrier ([6](#_ENREF_6)).

## Epi Info DE tool further improvements

Although Epi Info has different features that make it the tool of choice for DE in complex SR, further improvements are needed. The export function of Epi Info needs to be developed to allow the direct export of data in formats compatible with statistical software, such as SAS, Stata and R. Integration between Epi info and R is required to allow for direct discrepancy checking after the completion of the DE for a full or subset of the eligible studies. Finally, a module to track the DE progress and give an approximate time till completion can help meet the project timelines and determine whether the workload needs to be redistributed among the data extractors.

# Data comparison and adjudication

## Select – collapse - compare strategy

Data comparison can be done by selecting a subset of variables according to the level at which they were captured and then collapsing their values to unique values. The select and distinct functions of the R library "tidyverse" can be used. The data can then be fed into the compare_df function of the library "compareDF".

Repairs to the template dataset can use two processes depending on the discrepancy reason:

## Slice - bind strategy

This strategy can deal with discrepancies from an unmatched number of observations (rows), which occurs when one reviewer extracts more groups and/or outcomes than the other. These extra rows can be sliced and appended to the template dataset. To slice a subset of rows from one dataset and bind it to the other, the filter and bind_rows functions of the R library "tidyverse" can be used.

## Assignment of the adjudication value

Assignments of adjudication values replace values at the bottom of the dataset in the hierarchy with the correct values.

**References**

1. Centers for Disease Control and Prevention. Epi Info™ for Web & Cloud. Available at: https://www.cdc.gov/epiinfo/cloud.html. 2021.

2. Haddaway NR. Open Synthesis: on the need for evidence synthesis to embrace Open Science. Environmental Evidence. 2018;7(1):26.

3. Nieves E, Jones J. Epi Info™: Now an Open-source application that continues a long and productive "life" through CDC support and funding. The Pan African medical journal. 2009;2:6.

4. Westgate MJ. revtools: An R package to support article screening for evidence synthesis. Research Synthesis Methods. 2019;n/a(n/a).

5. Camp B, Mandivarapu JK, Ramamurthy N, Wingo J, Bourgeois AG, Cao X, et al. A new cross-platform architecture for epi-info software suite. BMC Bioinformatics. 2018;19(11):359.

6. Ma J, Otten M, Kamadjeu R, Mir R, Rosencrans L, McLaughlin S, et al. New frontiers for health information systems using Epi Info in developing countries: Structured application framework for Epi Info (SAFE). International Journal of Medical Informatics. 2008;77(4):219-2
